# Simultaneous Bilateral Primary Spontaneous Pneumothorax: A Case Report and a Review of the Literature

**DOI:** 10.1155/2019/6583842

**Published:** 2019-01-27

**Authors:** Jakrin Kewcharoen, Paul Morris, Chanavuth Kanitsoraphan, Hanh La, Narin Sriratanaviriyakul

**Affiliations:** ^1^University of Hawaii Internal Medicine Residency Programs, Honolulu, HI 96813, USA; ^2^Department of Surgery, The Queen's Medical Center, Honolulu, HI 96813, USA; ^3^Department of Internal Medicine, The Queen's Medical Center, Honolulu, HI 96813, USA

## Abstract

**Background:**

Simultaneous bilateral primary spontaneous pneumothorax (SBPSP) is an extremely rare and potentially fatal condition. Patients usually have no relevant medical conditions. Some cases, however, may have certain risk factors such as smoking, being young, and male gender. We reported a case of a healthy young male who presented with BPSP.

**Case Presentation:**

A 21-year-old man with a past medical history of well-controlled intermittent asthma presented with acute worsening shortness of breath overnight. Chest X-ray performed showed bilateral large pneumothorax with significantly compressed mediastinum. Chest tubes were placed bilaterally with immediate clinical improvement. However, the chest tubes continued to have an air leak without full lungs expansion. Computed tomography scan without contrast of the chest revealed subpleural blebs in both upper lobes. The patient underwent bilateral video-assisted thoracoscopic surgery (VATS) with apical bleb resection, bilateral pleurectomy, and bilateral doxycycline pleurodesis. Biopsy of the apical blebs and parietal pleura of both lungs were negative for any atypical cells suspicious for malignancy or Langerhans cell histiocytosis. The patient had been doing well six months following surgery with no recurrence of pneumothorax.

**Conclusion:**

SBPSP is a rare and urgent condition that requires prompt intervention. In a young patient without any underlying disease, surgical intervention, such as VATS, is relatively safe and can be considered early.

## 1. Introduction

Pneumothorax is a common medical condition defined by a presence of air or gas in the cavity in the pleural cavity. The incidence of spontaneous pneumothorax, the term used to call pneumothorax that arises by itself in the absence of trauma, was reported to be 1.2 to 28 cases per 100,000 [1]. Patients with spontaneous pneumothorax can present as either primary or secondary. The term primary spontaneous pneumothorax is used when there is no relevant medical condition found, although these patients may have certain risk factors, such as smoking, being young, and male gender. In contrast, the term secondary spontaneous pneumothorax is used when there is an underlying disease that is associated with the pneumothorax, such as lung tumor or chronic obstructive pulmonary disease (COPD) [2]. On rare occasions, spontaneous pneumothorax can present bilaterally. Simultaneous bilateral primary spontaneous pneumothorax (SBPSP) is an extremely rare presentation found in only 1% of all spontaneous pneumothorax [2]. This condition often causes significant respiratory distress and in some cases progresses to tension pneumothorax or death. Signs and symptoms of the SBPSP can be minimal initially thus requiring physicians to always be suspicious and aware of this disease [3]. Prompt management is needed to exclude tension pneumothorax and relieve the dyspnea. Unlike other types of pneumothorax, surgical intervention is indicated in SBPSP as it leads to better overall outcome compared to tube thoracostomy [1]. In this article, we report a case of a young male with SBPSP who was found to have bilateral lung blebs and eventually underwent bilateral blebs resection and bilateral pleurectomy.

## 2. Case Presentation

**Figure 1:**
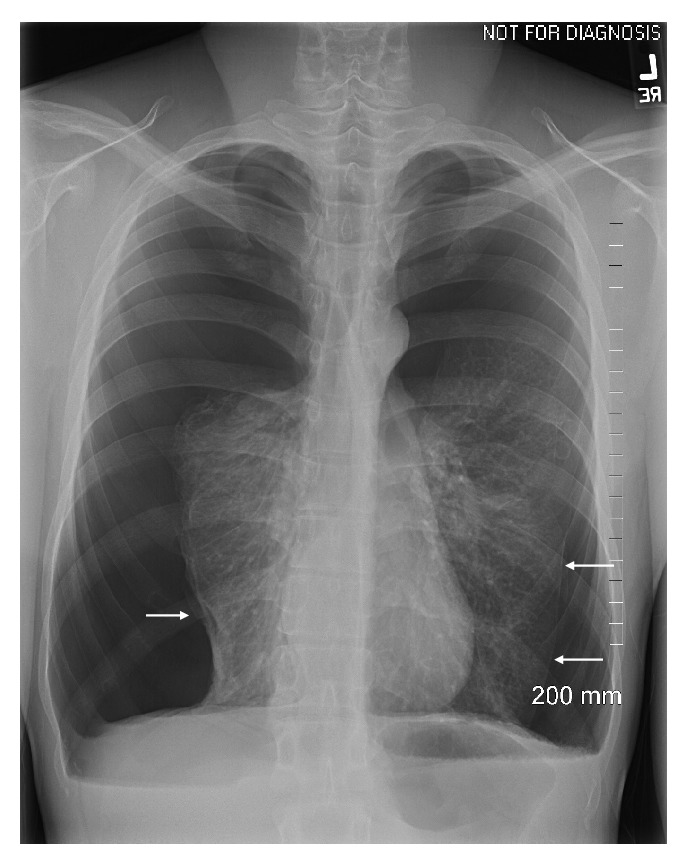
Chest radiograph with bilateral spontaneous pneumothorax at presentation. Pleural line is visible (arrow).

A 21-year-old man with a past medical history of asthma presented with acute worsening shortness of breath overnight with no identifiable trigger. The patient had experienced this symptom for three weeks although less severe initially. He also stated that the symptom was accompanied by cough, chest tightness, and pain across the anterior chest but could not clearly describe the characteristics of the pain. The difficult breathing was worsened with lying flat. He denied any history of smoking. On initial presentation, his vital signs included a temperature of 36.7°C, a blood pressure of 119/83 mmHg, a heart rate of 105 beats/min, respiratory rate of 18 breaths/min, and an oxygen saturation of 97% on room air. The patient was 170.2 cm tall and weighed 57.2 kg and BMI of 19.79 kg/m^2^. Physical examination revealed a distressed and ill-appearing male. Cardiopulmonary examination was notable for tachycardia, tachypnea, and decreased breath sounds in both upper lung fields. Laboratory results showed mild leukocytosis with a white blood cell count of 12.9 × 10^9^ cells/L, 72% neutrophils, and 16% lymphocytes. His hemoglobin level was 16.2 g/dl with a hematocrit of 48.6% and platelet count of 243 × 10^9^ cells/L. The blood biochemical profiles were unremarkable. Chest X-ray (CXR) (Figure 1) showed bilateral large pneumothorax (>2cm) with minimal bilateral pleural effusions and significantly compressed mediastinum.

**Figure 2:**
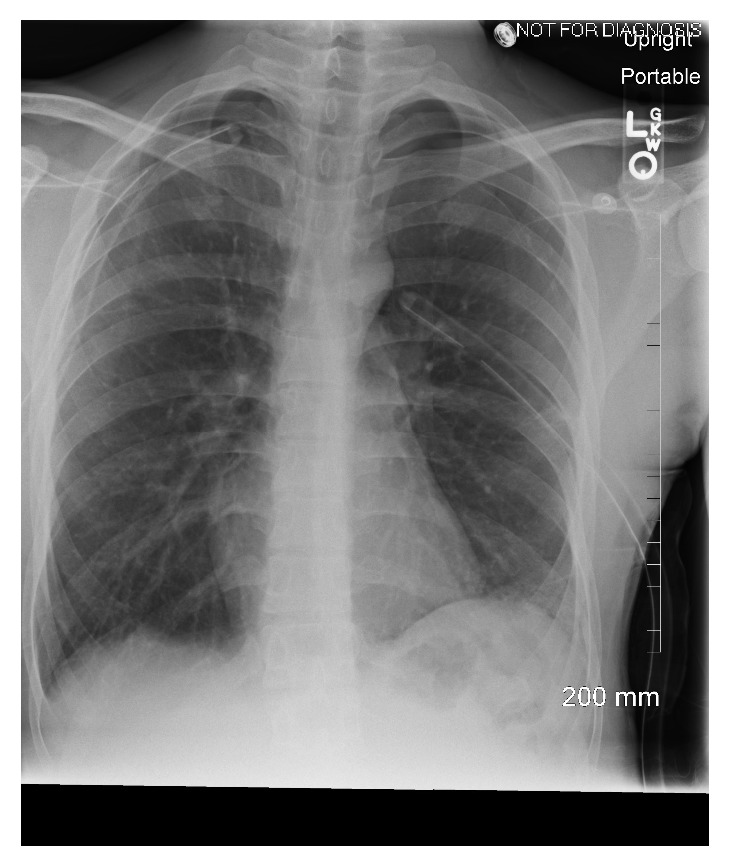
Bilateral lung re-expansion after bilateral chest tubes insertion.

**Figure 3:**
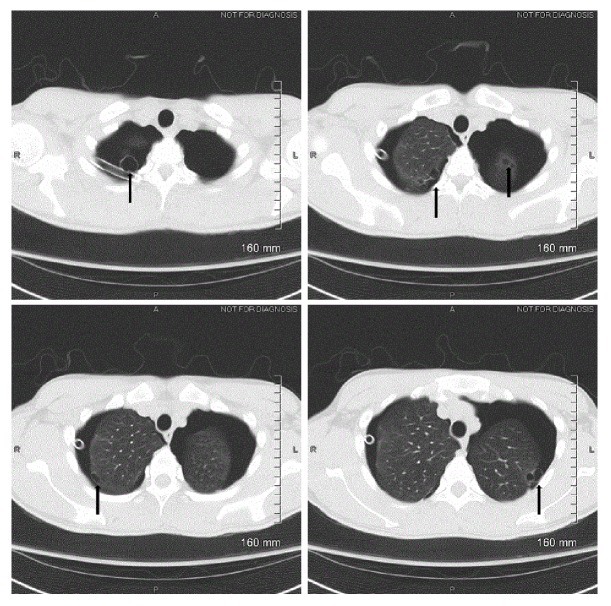
Chest computed tomography scan showing multiple subpleural blebs (arrow head).

A diagnosis of SBPSP was made. Chest tubes were placed bilaterally with immediate improvement in breathing and tachycardia. The right- and left-sided chest tubes drained serosanguinous fluids, 5 ml and 10 ml, respectively. Patient's clinical condition continued to improve and a follow-up CXR immediately following the procedures (Figure 2) showed a decrease of pneumothorax in both sides. However, during the hospital course, the chest tubes continued to have an air leak and the follow-up CXR continued to demonstrate residual pneumothorax without full lungs expansion. Computed tomography (CT) scan without contrast of the chest revealed subpleural blebs in both of the upper lobes (Figure 3). Due to continuous air leak without full lung expansion with conservative management, the patient was referred to thoracic surgery evaluation. One week later, our patient underwent bilateral VATS with apical bleb resection, bilateral pleurectomy, and bilateral doxycycline pleurodesis. Biopsy of the apical blebs and parietal pleura of both lungs showed fibrosis and granulation tissue, negative for any atypical cells suspicious for malignancy or Langerhans cell histiocytosis.

**Figure 4:**
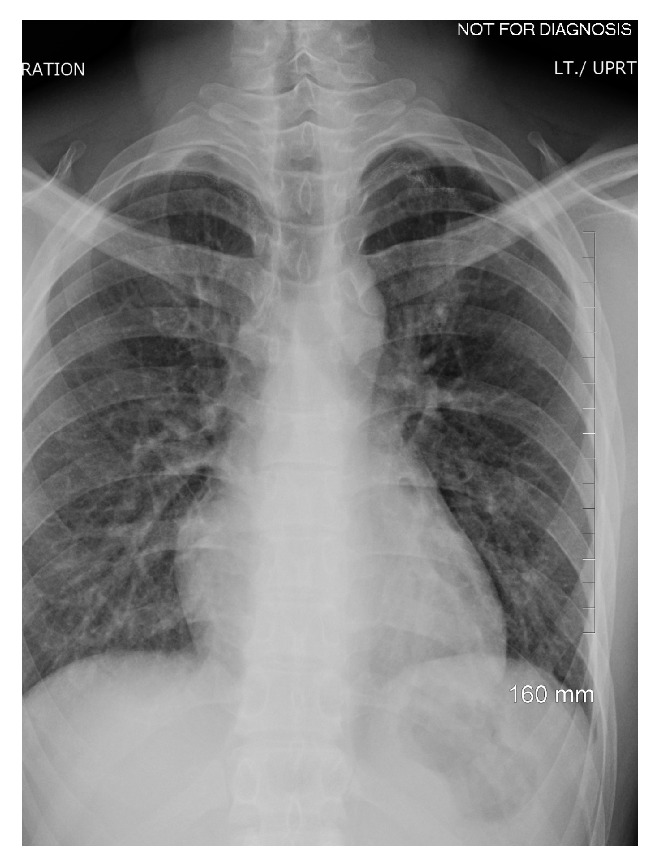
Chest radiograph during outpatient follow-up after the surgery.

On the subsequent outpatient follow-up visit, the patient had been doing well six months following surgery with no recurrence of pneumothorax (Figure 4).

## 3. Discussion

The diagnosis of pneumothorax is typically made by history and precise physical examination and may be followed by a CXR or chest ultrasound for confirmation [3]. The CXR provides a distinctive radiographic feature including a separation of visceral and parietal pleural line by a collection of gas, although a chest CT may later be performed to evaluate the etiology of the pneumothorax [3]. Signs and symptoms usually include acute or subacute shortness of breath, chest pain, and decreased breath sounds. However, if the amount of air in the pleural space is small, physical exam and chest X-ray may be normal. Spontaneous pneumothorax may rapidly progress to a fatal tension pneumothorax if left untreated. Thus, physicians should always have a low threshold for this diagnosis and work-up for this condition. On rare occasions, spontaneous pneumothorax can occur bilaterally and spontaneously and thus leads to a more serious disease spectrum.

The incidence of bilateral spontaneous pneumothorax (BSP) has been reported to be around 7.8-20% of all spontaneous pneumothorax [12]. Studies by Sayar et al. and Akcam et al. reported that approximately 58% and 68% of patients with BSP had causative underlying lung disease, respectively [1, 2]. Several medical conditions known to be associated with BSSP include pulmonary metastases, tuberculosis, Ehlers-Danlos syndrome, histiocytosis X, and COPD [2, 18–20]. Occasionally, BSSP can also occur from medical procedure or drugs [21, 22]. Bilateral spontaneous pneumothorax in a patient who had no underlying medical condition is called bilateral primary spontaneous pneumothorax (BPSP). Even though the definite mechanism is still not clear, rupture of the blebs in the lung is the most commonly known cause of the BPSP [1]. Among these patients, some would present as SBPSP, a rare presentation with limited data on incidence and prognosis. Some of the following characteristics may be seen in patients with SBPSP: (1) the patients tend to be younger, with low body mass index, (2) the patients may have a previous history of spontaneous unilateral pneumothorax, and (3) the size of pneumothorax is usually small [12, 23, 24]. In contrast, the patient in our case presented with significantly collapsed lungs and without any previous history of pneumothorax.

Due to a limited number of cases of SBPSP, there is no established consensus regarding treatment for this condition. Although the initial treatment of chest tube may provide a resolution of the disease, the other important consideration in patients with any type of pneumothorax is the risk of recurrence. Controversies exist on further surgical interventions once the lungs have achieved full expansion. According to the British Thoracic Society (BTS) guideline for the management of primary spontaneous pneumothorax, surgical intervention is indicated in some condition including SBPSP (Table 1) [4]. Despite the lack of data on recurrence rate and long-term prognosis, surgical intervention has also shown favorable results. Lee et al. performed bilateral VATS in 13 patients with SBPSP and reported 2 (7.7%) recurrence episodes [12]. However, the study by Cho et al., which reported the most cases of SBPSP, found the recurrence rate to be as high as 44% [7]. In this study, Cho et al. performed ipsilateral transmediastinal approach VATS in approximately half of the patients and traditional bilateral sequential approach in the other half. Nevertheless, postoperative recurrence was not significantly different between the two groups. Thus, Cho et al. suggested that this may be due to limited surgeon's experience of SBPSP cases.

We conducted a literature review of SBPSP patients who underwent VATS. We identified 14 studies/case reports from 2002 to 2018 with a total of 81 patients with SBPSP who underwent VATS. From the available data, most of the patients were male (98.3%) and young. Every patient underwent bilateral VATS except 3 patients in the study by Akcam et al. where they initially had unilateral VATS. The recurrence rate was available from 9 articles. For patients who underwent bilateral VATS, the overall recurrence rate was 27.3%. However, it is important to note that the number is largely influenced by one relatively large study by Cho et al. that showed the recurrence rate of 44% [7]. All included studies are summarized in Table 2. Unfortunately, we were not able to find enough cases and studies that were managed conservatively without surgical interventions to compare the rate of recurrence. Based on a recent study published by Akcam et al., all patients with SBPSP who only had a chest tube placed on the initial episode subsequently recurred and eventually required a surgical operation [1]. These patients had no further recurrence on follow-up.

In conclusion, SBPSP is a rare urgent medical condition that requires prompt treatment. In a young patient without any underlying disease, surgical intervention, such as VATS, is relatively safe and can be considered early.

## Figures and Tables

**Table 1:** Accepted indications for surgical intervention [4].

**Accepted indications for surgical advice should be as follows:**

Second ipsilateral pneumothorax
First contralateral pneumothorax
Synchronous bilateral spontaneous pneumothorax
Persistent air leak (despite 5–7 days of chest tube drainage) or failure of lung re-expansion
Spontaneous haemothorax
Professions at risk (eg, pilots, divers)
Pregnancy

**Table 2:** Summary of the reported cases of simultaneous bilateral primary spontaneous pneumothorax treated with surgical intervention.

First author	Year	N	Mean age (Years)	Male (%)	Surgical intervention	Mean follow-up (months)	Recurrence (n)
Akcam [1]	2018	5	22.4	5 (100)	Single-staged VATS	N/A	3*∗*
Aye [5]	2002	4	21.5	4 (100)	Single-staged VATS	N/A	0
Chen [6]	2008	4	22.1	4 (100)	Single-staged VATS	45.6	1^¥^
Cho [7]	2017	25^£^	16.3	24 (96)	Single-staged VATS	62	11*∗∗*
Hatzigeorgiadis [8]	2014	1	20	1 (100)	Single-staged VATS	36	0
Kim [9]	2017	2	17, 18	2 (100)	Single-staged VATS	24, 19	0
Guo [10]	2016	4	N/A	N/A	Single-staged VATS	N/A	N/A
Lang-Lazdunski [11]	2003	3	N/A	N/A	Single-staged VATS	N/A	N/A
Lee [12]	2008	13	20.9	13 (100)	Single-staged VATS	44.4	2
Okubo [13]	2014	1	16	1 (100)	Single-staged VATS	N/A	0
Sachithanandan [14]	2012	2	26, 17	2 (100)	Single-staged VATS	N/A	1
Soccorso [15]	2015	5	N/A	N/A	Single-staged VATS	N/A	N/A
Watanabe [16]	2004	1	23	1 (100)	Single-staged VATS	N/A	0
Yueng [17]	2016	11	N/A	N/A	Single-staged VATS	N/A	N/A

VATS: video-assisted thoracoscopic surgery

*∗*: Two patients had unilateral VATS and tube thoracostomy on the other side. The other patient had unilateral VATS without any intervention on the other lung. All recurrence occurred in the un-operated lung.

¥: Prolonged air-leakage (>7 days) post-operation

£: Eleven patients underwent ipsilateral transmediastinal approach instead of bilateral sequential approach

*∗∗*: Five recurrences were from the ipsilateral transmediastinal approach group while the other six recurrences were fromthe traditional bilateral sequential approach group
